# Increased IgG4-Positive Plasma Cells in Granulomatosis with Polyangiitis: A Diagnostic Pitfall of IgG4-Related Disease

**DOI:** 10.1155/2012/121702

**Published:** 2012-04-11

**Authors:** Sing Yun Chang, Karina Keogh, Jean E. Lewis, Jay H. Ryu, Eunhee S. Yi

**Affiliations:** ^1^Division of Anatomic Pathology, Mayo Clinic, Rochester, MN 55905, USA; ^2^Division of Pulmonary and Critical Care Medicine, Mayo Clinic, Rochester, MN 55905, USA

## Abstract

Granulomatosis with polyangiitis (Wegener's) (GPA) may mimic IgG4-related disease (IgG4-RD) on histologic examination of some biopsies, especially those from head and neck sites. IgG4 immunostain is often performed in this context for differential diagnosis with IgG4-RD. However, the prevalence of IgG4+ cells in GPA has not been explored. We examined the IgG4+ cells in 26 cases confirmed as GPA by a thorough clinical and pathologic assessment. Twenty-six biopsies consisted of 14 sinonasal/oral cavity/nasopharynx, 7 orbit/periorbital, 3 lung/pleura, 1 iliac fossa/kidney, and 1 dura specimens. Eight of 26 (31%) biopsies revealed increased IgG4+ cells (>30/HPF and >40% in IgG4+/IgG+ ratio). The IgG4+ cells and IgG4+/IgG+ ratio ranged 37–137/hpf and 44–83%, respectively. Eight biopsies with increased IgG4+ cells were from sinonasal (*n* = 4) or orbital/periorbital (*n* = 4) sites. In conclusion, increased IgG4+ cells are not uncommonly seen in sinonasal or orbital/periorbital biopsies of GPA, which could pose as a diagnostic pitfall.

## 1. Introduction

Granulomatosis with polyangiitis (Wegener's) (GPA) is an immune-mediated systemic necrotizing vasculitis often affecting the upper respiratory tract, lung, and kidney [1]. Involvement of GPA is limited to the upper respiratory tract and/or the lung in some cases although virtually anybody site can be involved in GPA such as the eye, skin, joints, heart, and the central nervous system [2]. Necrotizing vasculitis and irregular basophilic parenchymal necrosis with associated palisading granuloma comprise the main histologic characteristics of GPA. Also, neutrophilic microabscesses and fibrosis are commonly found in a background mixed inflammatory infiltrate composed of neutrophils, lymphocytes, plasma cells, multinucleated giant cells, and macrophages [1, 2].

On histologic examination, GPA can mimic IgG4-related disease (IgG4-RD) since the inflammatory background in GPA may be rich in plasma cells and accompanied by fibrosis and/or obliterated blood vessels as in IgG4-RD [1, 3, 4]. Some biopsies of GPA cases (especially from the upper respiratory tract and orbit) may lack classic morphologic features such as necrotizing vasculitis, parenchymal necrosis, and palisading granuloma [5, 6]. IgG4 immunostain is now often performed in this context for evaluating the possibility of IgG4-RD. However, the prevalence of IgG4-positive (IgG4+) cells in GPA has not been widely reported in the literature. Therefore, we sought to assess the prevalence of IgG4+ cells in GPA cases that have been confirmed by a thorough clinical and pathologic assessment.

## 2. Materials and Methods

### 2.1. Case Selection

 Cases with the diagnosis of GPA were identified via electronic search from our surgical pathology file. We initially retrieved 36 biopsies from various body sites obtained during a period of 1999–2011. The sites of biopsies included sinus/nasopharynx (*n* = 16), oral cavity (*n* = 1), orbital region (*n* = 11), lung/pleural (*n* = 6), kidney (*n* = 1), and dura (*n* = 1). Glass slides from each biopsy were reviewed and reassessed for the following histologic features: geographic necrosis, necrotizing granulomas, vasculitis, multinucleated giant cells, microabscesses, and fibrosis. Based on these histologic parameters, each case was scored as to the confidence of histopathologic diagnosis of GPA as follows: 0 = nondiagnostic biopsy or not GPA with some findings against the diagnosis of GPA such as infection, 1 = nonspecific (one feature present); 2 = suggestive of GPA (two features present), or 3 = consistent with GPA (three or more features present). A definitive clinicopathologic diagnosis of GPA was made if a case had met the modified clinical criteria of the American College of Rheumatology (ACR) for GPA [7, 8] (modified to include assessment of ANCA status) and a pathology diagnostic score of 1, 2, or 3. Twenty-six of the initial 36 cases were confirmed as the diagnosis of GPA and retained for this study.

### 2.2. Immunohistochemistry

 Immunohistochemical staining for IgG4 and IgG was performed on all 26 cases using the DAKO dual multimer system or DAKO advance 2 stops multimer system (DAKO, Carpinteria, CA, USA). Monoclonal IgG4 antibody (clone HP6025, dilution 1 : 100; Zymed, San Francisco, CA, USA) and polyclonal rabbit antihuman IgG antibody (dilution 1 : 15,000, DAKO, Carpinteria, CA, USA) were used for the study.

### 2.3. Quantitation

 IgG4+ and IgG+ cells were counted using the following steps.

1. Areas of highest density of IgG4+ plasma cells “hot-spot” were identified at low power magnification.
2. Using 40x objective lens (Olympus BX50 microscope, 40x objective, 10x eye piece, Olympus DP70 camera, on-screen capture field diameter of 0.34 mm and field area of 0.0884 mm^2^), three high-power fields (HPFs) of IgG4+ hot spots, and their corresponding areas of IgG immunostain were photographed.
3. All photographs were printed for a manual counting of the absolute number of IgG4+ and IgG+ plasma cells in each HPF.
4. Averages of IgG4+ and IgG+ cells in three HPFs were used to determine the IgG4+ cell count and IgG4+/IgG+ ratio for each case. Increase in IgG4+ cells was defined as the average IgG4+ cells greater than 30 per HPF with the IgG4+/IgG+ cell ratio greater than 40%.

### 2.4. Serum C-Reactive Protein and Immunoglobulin Levels

 Patients' medical records from our institution were reviewed for their serum levels of C-reactive protein (CRP) and immunoglobulin subclasses.

## 3. Results

**Figure 1:**
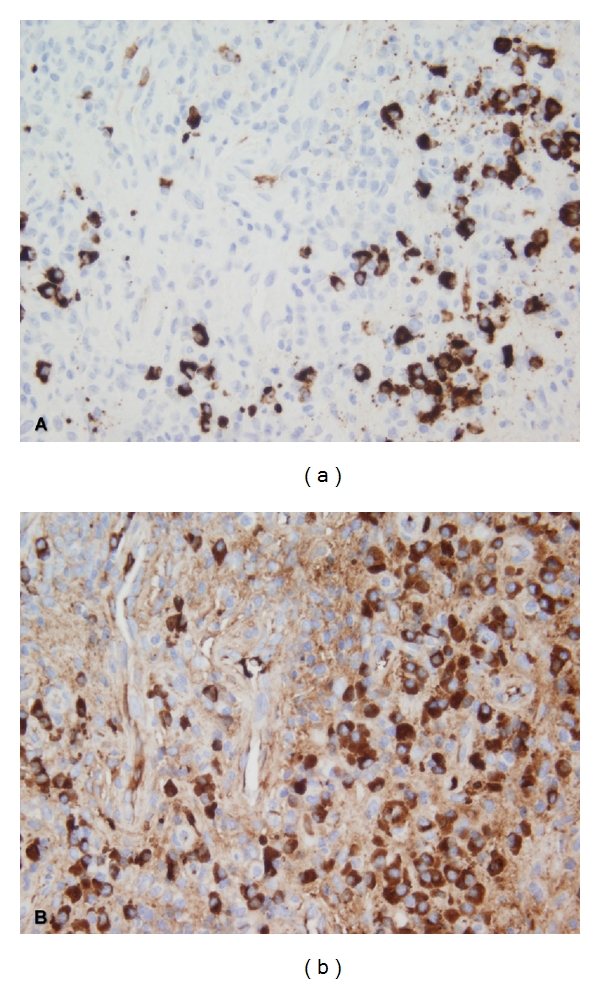
(a) Increased IgG4-positive cells with a high IgG4+/IgG+ ratio. (b) IgG+ cells from corresponding hotspot (immunohistochemistry, 400x original magnification).

**Figure 2:**
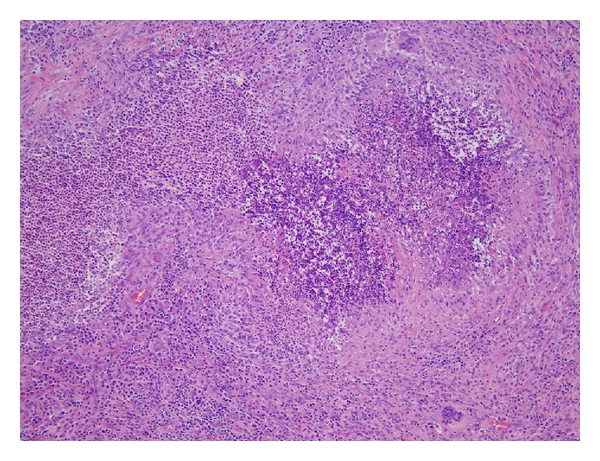
Parenchymal necrosis with palisading granuloma, necrotizing vasculitis, and mixed inflammatory infiltrates, characteristic of GPA (hematoxylin and eosin, 100x original magnification).

Eight of 26 biopsies (31%) showed increased IgG4+ cells with an average IgG4+ cell count and an IgG4+/IgG+ cell ratio ranging from 37 to 137/HPF and from 44 to 83%, respectively (Figure 1). These 8 biopsies were from sinonasal (*n* = 4) and orbital/periorbital (*n* = 4). The IgG4+ cell count ranged from 0 to 28/HPF in the remaining 18 biopsies that were from sinonasal/oral cavity/nasopharynx (*n* = 10), orbit/periorbital (*n* = 3), lung/pleura (*n* = 3), iliac fossa/kidney (*n* = 1), and dura (*n* = 1). The IgG4+ cell counts and IgG4/IgG ratios in the cases without increase in IgG4+ cells ranged from 0 to 28 per HPF and from 0 to 39%, respectively (Table 1). There was no significant difference in the distribution of age at diagnosis or gender between the cases with and without increased IgG4+ cells. Elevated titers of antineutrophil cytoplasmic autoantibodies (ANCA) were present in 25 of 26 cases in this study. All 8 cases with increased IgG4+ cells were positive for ANCA; seven cases were C-ANCA positive, 6 of which were also positive for proteinase 3 (PR-3) ANCA by ELISA. One case was positive for P-ANCA confirmed as myeloperoxidase (MPO) ANCA by ELISA. On histopathologic evaluation, most cases showed characteristic histologic findings of GPA (Figure 2); 2 of 8 cases were graded as pathologic score 3, 2 as score 2, and the remaining 4 as score 1. Details are summarized in Table 1.

The 26 biopsies in this study were obtained from 23 patients. The status of serum CRP level was assessed at the time of the biopsy in 18 patients, and it was elevated in 15 of them (83%) (Table 1). Immunoglobulin levels were evaluated in only three patients (case 7, 9, and 17) and did not reveal polyclonal hypergammaglobulinemia. The serum IgA and IgG4 levels in these three patients were within normal range. The eight biopsies with increased IgG4+ cells were taken from seven patients, five of whom had elevated CRP levels (Table 1). The two patients with normal CRP levels demonstrated classic histopathologic features including geographic necrosis, palisading granulomas, vasculitis, or microabscesses, which supported the clinicopathologic diagnosis of GPA despite the normal CRP level.

## 4. Discussion

In this study, we sought to determine the prevalence of increased IgG4+ plasma cells in GPA in order to address the role of this finding in the differential diagnosis with IgG4-RD. We believe that this is the largest series of GPA cases to examine IgG4+ cells with application of current criteria and method for evaluation of increased IgG4+ cells in the setting of IgG4-RD. An increase in IgG4+ cells in GPA has been suggested in a recent study by Vaglio et al. [9] in which they reviewed tissue IgG4/IgG ratio in 10 GPA cases along with 9 cases with the Churg-Strauss syndrome (CSS) and 22 cases with chronic sinusitis. However, they did not provide the details on the counting method or results of IgG4+ cells.

In our study, we applied the current criteria and methods in counting IgG4+ cells, which should provide useful and important information in routine diagnostic surgical pathology practice. Also, we made an extra effort to ensure the diagnosis of GPA by a thorough clinicopathologic evaluation and only included the cases with irrefutable diagnosis of GPA. All but one case showed positivity for C- or P-ANCA confirmed with ELISA for PR3 or MPO; the single ANCA negative case demonstrated definite histopathologic findings (pathologic diagnosis score 3) as well as clinical features for GPA, which supported our diagnosis of GPA. Moreover, all 8 cases with increased IgG4+ cells in this study were positive for ANCA, which reiterated our point.

The cutoff number of increased IgG4+ plasma cells has not been well established, and many studies have suggested different cutoff points. Kamisawa et al. and Zhang et al. have used a cutoff point of >30 per HPF [10, 11] (40x objective lens) while Deshpande et al. and Dhall et al. used >50 HPF (20x or 40x objectives, resp.), and they have reached high sensitivity and specificity in diagnosing autoimmune pancreatitis [12, 13]. In addition to this variable thresholds, the method for counting IgG4+ cells has not been well standardized. We used the same method as in our previous study [14] by enumerating cells on printed images and using averages of three “hot spots” in order to ensure accuracy and reproducibility. We also applied a higher threshold for increased IgG4+ cells by using both the IgG4+ cell count per HPF at >30 and the ratio of IgG4+/IgG+ cells at >40%. Cheuk et al. have proposed that both absolute number of IgG4+ cell higher than 50 per HPF and IgG4+/IgG+ ratio greater than 40% should be present in order to make the diagnosis of IgG4-RD involving lymph nodes and other extranodal sites [3]. Although our cutoffs for IgG4+ status may be slightly lower than the histologic criteria proposed by Cheuk et al. (which also used averages of three different HPFs), the latter used a wider field area than the one in our study (0.196 mm^2^ versus 0.088 mm^2^, resp.). Therefore, the threshold used in this study would be comparable to the one of the most stringent ones in the literature.

The most recent comprehensive diagnostic criteria for IgG4-RD published by Umehara et al. included two major components: (1) serum IgG4 concentration of >135 mg/dL and (2) >10 IgG4+ plasma cells/HPF with a ratio of IgG4+/IgG+ cells >40% [15]. IgG4 serum level was not increased in the 3 cases tested in our study. Instead, most of our GPA cases showed elevated serum CRP which is unusual for IgG4-RD. Based on their criterion for IgG4+ cell count as >10, however, potentially additional five cases in our study would have been considered to have sufficiently increased IgG4+ cells, which will make 50% of our GPA cases with increased IgG4+ cells.

Positive ANCA has been reported in patients with Grave's disease receiving antithyroidal medication such as propylthiouracil (PTU) [16, 17] and may even mimic GPA clinically [18]. However, none of our patients had a history of either Graves's disease or treatment with PTU. The possibility of positive ANCA in patients with IgG4-RD has not been addressed in previous studies, and whether this can occur has yet to be determined. A further study with testing for ANCA in IgG4-RD would be needed to answer this question.

Previous studies have reported the presence of inflammatory infiltrates rich in IgG4+ plasma cells in clinical settings other than IgG4-RD [19–21], usually as an isolated finding. In kidneys, several studies have highlighted a frequent association of IgG4+ plasma cell-rich infiltrates with the glomerular lesions that are typically seen in ANCA-associated angiitis, namely, pauci-immune necrotizing and crescentic glomerulonephritis [19, 20]. In midst of all these findings, questions regarding the nature of relationship between IgG4+ plasma cells and GPA can arise. Interestingly, there have been some data suggesting that ANCA of IgG4 subclass possibly plays a role in the pathogenesis of ANCA-related small vessel vasculitis [22–24].

The predominance of IgG4 as well as IgG1 subclasses of ANCA was first reported in patients with GPA and other clinically related disorders by Brouwer et al. in 1991 [22]. Holland et al. later suggested a possible pathogenic role for the IgG4 subclass in GPA [23]. *In vitro*, ANCA activated neutrophils by colligating PR3 and Fc*γ*RIIa/IIIb receptors [23]. ANCA are predominantly of the IgG isotype, and IgG1, IgG3, and IgG4 subclasses are particularly represented. IgG4 subclass isolated from ANCA-positive sera demonstrated varying abilities to stimulate release of superoxide, which was unrelated to PR3-ANCA titer, neutrophil donor used in their *in vitro* test, or neutrophil Fc*γ*RI expression [23]. This study suggested that IgG4 was capable of activating neutrophils via constitutively expressed Fc*γ*RIIa/IIIb or colligation of other unidentified cell surface molecules [23]. Liu et al. have reported that MPO-ANCA IgG4 subclass might play a role in the development of GPA [25]. They reported that the titers of anti-MPO IgG4 subclass in patients with GPA was significantly higher than those with microscopic polyangiitis (MPA). The MPO-ANCA in GPA and MPA might recognize overlapping but different epitopes on native MPO molecule. The difference in immunological characteristics of MPO-ANCA might have contributed to different disease entities such as GPA and MPA. Another recent study reported that serum IgG4 levels were markedly elevated in active CSS and also correlated with the disease activity and the number of involved organs [9].

 Given these serological and immunological findings, one can postulate a possible role of tissue infiltrating IgG4+ cells in the pathogenesis of GPA. However, the underlying cause or precise mechanism for increased IgG4+ cells in the tissue as seen in some of our GPA cases has not been completely elucidated and further study would be needed in the future. Also, whether there is any pathogenetic relationship between GPA and IgG4-RD is not entirely clear. Although there have been studies reporting elevated IgG4 in the setting of ANCA-associated systemic diseases, no study demonstrating elevated ANCA in IgG4-RD exists in the English literature to our knowledge. Our anecdotal experiences also indicate that the ANCA is generally not elevated in IgG4-RD.

In conclusion, one should be aware of the fact that IgG4+ cells can be remarkably increased in biopsies of GPA of the sinonasal and orbital/periorbital regions. Since the morphologic and clinical manifestations of GPA and IgG4-RD may overlap, it could be a significant diagnostic pitfall in the differential diagnosis of these two entities. Further study is needed to confirm our observation in a larger number of cases, and a further exploration of potential pathogenetic relationship between GPA and IgG4-RD might be of interest as well.

## Figures and Tables

**Table 1:** Clinical features, immunohistochemical findings, ANCA details, and CRP level.

Case no.	Sex	Age at Dx	Biopsy site	IgG4 average	IgG4/IgG ratio	Path Dx score	Clinical Dx (modified ACR criteria)	ANCA (+)	P-ANCA	MPO	C-ANCA	PR3	CRP
1	M	74	Nasal septum	28	87%	3	Generalized	y	n	n	y	y	High
2	F	83	Nasal sinus	20	63%	3	Limited	y	n	n	y	y	High
3	M	54	Nasal septum	62	59%	3	Generalized	y	n	n	y	y	High
4	F	59	Lung RUL RML	27	28%	3	Limited	y	y	y	n	n	NA
5	F	19	Oral cavity	6	19%	3	Generalized	y	n	n	y	y	NA
6	F	18	Nasal sinus	11	79%	1	Generalized	y	y	y	n	n	NA
7*	M	47	Orbit	49	58%	2	Generalized	y	n	n	y	y	High*
8*	M	47	Nasal septum	4	19%	1	Generalized	y	n	n	y	y	*
9**	F	31	Lung, left, pneumonectomy	16	33%	3	Generalized	y	n	n	y	y	NA**
10**	F	31	Kidney	26	39%	3	Generalized	y	n	n	y	y	**
11	M	52	Bilateral orbital mass	1	1%	3	Generalized	n	n	n	n	n	High
12	M	31	Nasal cavity	13	32%	2	Limited	y	n	n	y	y	High
13	F	16	Nasal cavity	1	7%	1	Limited	y	n	n	y	y	High
14	F	47	Orbital soft tissue	24	53%	2	Generalized	y	y	n	y	y	High
15	F	23	Nasopharynx	18	57%	2	Generalized	y	n	n	y	y	High
16	M	44	Nasal cavity	0	0%	2	Generalized	y	n	n	y	y	High
17***	M	36	Nasal cavity	43	47%	1	Generalized	y	n	n	y	y	Normal***
18***	M	36	Eyelid/orbital fat	53	81%	2	Generalized	y	n	n	y	y	***
19	F	64	Nasal cavity	8	13%	2	Generalized	y	y	n	n	n	High
20	F	13	Orbit	15	30%	1	Generalized	y	y	n	n	n	Normal
21	F	23	Orbital mass	69	83%	3	Generalized	y	n	n	y	y	Normal
22	F	71	Periorbital	139	82%	1	Generalized	y	y	y	n	n	High
23	F	31	Nasal cavity	55	73%	1	Generalized	y	n	n	y	y	High
24	F	69	Dura parietooccipital	5	10%	2	Generalized	y	y	n	n	n	High
25	F	40	Pleura	9	34%	3	Generalized	y	n	n	y	y	NA
26	F	49	Sinonasal	37	44%	1	Limited	y	n	n	y	y	High

*, **, *** denote the same patients. Dx: diagnosis; Path: pathology; ACR: American College of Rheumatology; ANCA: antineutrophil cytoplasmic antibody; PR3: proteinase 3; MPO: myeloperoxidase; NA: not available; CRP: c-reactive protein.

## References

[1] Leslie KO, Wick MR (2011). *Practical Pulmonary Pathology: A Diagnostic Approach*.

[2] Yi ES, Colby TV (2001). Wegener's granulomatosis. *Seminars in Diagnostic Pathology*.

[3] Cheuk W, Chan JK (2010). IgG4-related sclerosing disease: a critical appraisal of an evolving clinicopathologic entity. *Advances in Anatomic Pathology*.

[4] Smyrk TC (2011). Pathological features of IgG4-related sclerosing disease. *Current Opinion in Rheumatology*.

[5] Kalina PH, Lie JT, Campbell RJ, Garrity JA (1992). Diagnostic value and limitations of orbital biopsy in Wegener’s granulomatosis. *Ophthalmology*.

[6] Devaney KO, Travis WD, Hoffman G, Leavitt R, Lebovics R, Fauci AS (1990). Interpretation of head and neck biopsies in Wegener’s granulomatosis. A pathologic study of 126 biopsies in 70 patients. *American Journal of Surgical Pathology*.

[7] (2002). Design of the wegener's granulomatosis etanercept trial (WGET). *Controlled Clinical Trials*.

[8] Stone JH (2003). Limited versus severe Wegener’s granulomatosis: baseline data on patients in the Wegener’s granulomatosis etanercept trial. *Arthritis and Rheumatism*.

[9] Vaglio A, Strehl JD, Manger B (2012). IgG4 immune response in Churg-Strauss syndrome. *Annals of the Rheumatic Diseases*.

[10] Kamisawa T, Funata N, Hayashi Y (2003). A new clinicopathological entity of IgG4-related autoimmune disease. *Journal of Gastroenterology*.

[11] Zhang L, Notohara K, Levy MJ, Chari ST, Smyrk TC (2007). IgG4-positive plasma cell infiltration in the diagnosis of autoimmune pancreatitis. *Modern Pathology*.

[12] Deshpande V, Chicano S, Finkelberg D (2006). Autoimmune pancreatitis: a systemic immune complex mediated disease. *American Journal of Surgical Pathology*.

[13] Dhall D, Suriawinata AA, Tang LH, Shia J, Klimstra DS (2010). Use of immunohistochemistry for IgG4 in the distinction of autoimmune pancreatitis from peritumoral pancreatitis. *Human Pathology*.

[14] Shrestha B, Sekiguchi H, Colby TV (2009). Distinctive pulmonary histopathology with increased IgG4-positive plasma cells in patients with autoimmune pancreatitis: report of 6 and 12 cases with similar histopathology. *American Journal of Surgical Pathology*.

[15] Umehara H, Okazaki K, Masaki Y (2011). Comprehensive diagnostic criteria for IgG4-related disease (IgG4-RD), 2011. *Modern Rheumatology*.

[16] Khosroshahi A, Stone JH (2011). A clinical overview of IgG4-related systemic disease. *Current Opinion in Rheumatology*.

[17] Gunton JE, Stiel J, Clifton-Bligh P, Wilmshurst E, McElduff A (2000). Prevalence of positive anti-neutrophil cytoplasmic antibody (ANCA) in patients receiving anti-thyroid medication. *European Journal of Endocrinology*.

[18] Pillinger M, Staud R (1998). Wegener’s granulomatosis in a patient receiving propylthiouracil for Graves’ disease. *Seminars in Arthritis and Rheumatism*.

[19] Raissian Y, Nasr SH, Larsen CP (2011). Diagnosis of IgG4-related tubulointerstitial nephritis. *Journal of the American Society of Nephrology*.

[20] Houghton DC, Troxell ML (2011). An abundance of IgG4 plasma cells is not specific for IgG4-related tubulointerstitial nephritis. *Modern Pathology*.

[21] Strehl JD, Hartmann A, Agaimy A (2011). Numerous IgG4-positive plasma cells are ubiquitous in diverse localised non-specific chronic inflammatory conditions and need to be distinguished from IgG4-related systemic disorders. *Journal of Clinical Pathology*.

[22] Brouwer E, Tervaert JW, Horst G (1991). Predominance of IgG1 and IgG4 subclasses of anti-neutrophil cytoplasmic autoantibodies (ANCA) in patients with Wegener’s granulomatosis and clinically related disorders. *Clinical and Experimental Immunology*.

[23] Holland M, Hewins P, Goodall M, Adu D, Jefferis R, Savage CO (2004). Anti-neutrophil cytoplasm antibody IgG subclasses in Wegener’s granulomatosis: a possible pathogenic role for the IgG4 subclass. *Clinical and Experimental Immunology*.

[24] Hussain A, Pankhurst T, Goodall M (2009). Chimeric IgG4 PR3-ANCA induces selective inflammatory responses from neutrophils through engagement of Fcgamma receptors. *Immunology*.

[25] Liu LJ, Chen M, Yu F, Zhao MH, Wang HY (2008). IgG subclass distribution, affinity of anti-myeloperoxidase antibodies in sera from patients with Wegener’s granulomatosis and microscopic polyangiitis. *Nephrology*.

